# The legacy of Prof Rinaldo Bellomo for critical care and scientific publication: an editorial tribute from Critical Care Science

**DOI:** 10.62675/2965-2774.20250179

**Published:** 2025-05-04

**Authors:** Ary Serpa, Jorge Ibrain Figueira Salluh

**Affiliations:** 1 Hospital Israelita Albert Einstein Department of Critical Care Medicine São Paulo SP Brasil Department of Critical Care Medicine, Hospital Israelita Albert Einstein - São Paulo (SP), Brasil.; 2 Brazilian Research in Intensive Care Network São Paulo SP) Brazil Brazilian Research in Intensive Care Network (BRICNet) - São Paulo (SP), Brazil.; 3 Monash University School of Public Health and Preventive Medicine Australian and New Zealand Intensive Care Research Centre Melbourne Australia Australian and New Zealand Intensive Care Research Centre, School of Public Health and Preventive Medicine, Monash University - Melbourne, Australia.; 4 Instituto D’Or de Pesquisa e Ensino Rio de Janeiro RJ Brazil Instituto D’Or de Pesquisa e Ensino - Rio de Janeiro (RJ), Brazil.

The global intensive care community mourns the loss of a visionary. Professor Rinaldo Bellomo's passing leaves an irreplaceable void, but also gives us the opportunity of learning from his imense legacy. Professor Bellomo was not only one of the most prolific and cited researchers in the history of critical care, he was also a tireless advocate for collaboration, mentorship, and scientific integrity. His academic output is impressive: more than 2,000 scientific publications, countless high-impact clinical trials, and foundational work in acute kidney injury, fluid resuscitation, and sepsis. However, statistics alone cannot capture the full scope of his influence.

Rinaldo's legacy is deeply linked to the evolution of critical care publishing. He had a clear vision: that science should be impactful, accessible, collaborative, and global in scope. He believed in the strength of evidence and networks, the value of mentoring, and the power of scientific exchange. This ethos informed his long-standing support for Critical Care Science. As a board member, he actively contributed to the discussion of how our mission could be a bridge between high-quality research and the clinical realities of low- and middle-income countries, especially considering the current publication landscape.^(1,2)^

Through his encouragement and enthusiasm, a recent partnership between Critical Care Science and Critical Care and Resuscitation, where he served as Editor-in-Chief, was born.^(3)^ He believed that journals should not compete for prestige but unite to amplify impact. Together, we began to build a pipeline for knowledge exchange between Latin America, Australasia, and beyond. He saw these collaborations as essential to a more inclusive and evidence-based critical care community.

Prof. Bellomo's work extended internationally and was relevant for Brazilian intensive care unit researchers and networks like BRICNet. He was an inspiring and generous ally through his belief in the potential of Latin American research. Beyond science, Rinaldo was a builder of people and ideas who embodied a rare combination of intellectual rigor and human warmth, and he was always driven by a deep commitment to better care for critically ill patients. As we reflect on his contributions, Critical Care Science reaffirms its commitment to the principles he stood for: scientific excellence, collaboration across borders, and the nurturing of young researchers. His legacy challenges us to be more ambitious, inclusive, and collaborative.

Rinaldo Bellomo was, and will remain, a guiding light in critical care medicine. His work changed practice, his mentorship changed lives, and his vision helped shape a global community of inquiry and care. We are honored to have worked alongside him and challenged to carry forward the values he held so dearly. On behalf of the editorial board of Critical Care Science, we extend our heartfelt condolences to his family, colleagues, and the many friends he inspired worldwide. His impact endures in every journal issue, every citation, and every researcher he impacted along the way.
